# Motor and psychosocial impact of robot-assisted gait training in a real-world rehabilitation setting: A pilot study

**DOI:** 10.1371/journal.pone.0191894

**Published:** 2018-02-14

**Authors:** Cira Fundarò, Anna Giardini, Roberto Maestri, Silvia Traversoni, Michelangelo Bartolo, Roberto Casale

**Affiliations:** 1 Neurophysiopathology Unit, Istituti Clinici Scientifici Maugeri, IRCSS, Montescano (PV), Italy; 2 Psychology Unit, Istituti Clinici Scientifici Maugeri, IRCSS, Montescano (PV), Italy; 3 Department of Biomedical Engineering, Istituti Clinici Scientifici Maugeri, IRCSS, Montescano (PV), Italy; 4 Habilita Care & Research Hospitals, Neurorehabilitation Unit and Department of Advanced Technology Rehabilitation & Pain Rehabilitation Units, Zingonia di Ciserano (BG), Italy; University of Illinois at Urbana-Champaign, UNITED STATES

## Abstract

In the last decade robotic devices have been applied in rehabilitation to overcome walking disability in neurologic diseases with promising results. Robot assisted gait training (RAGT) using the Lokomat seems not only to improve gait parameters but also the perception of well-being. Data on the psychosocial patient-robot impact are limited, in particular in the real-world of RAGT, in the rehabilitation setting. During rehabilitation training, the Lokomat can be considered an “assistive device for movement”. This allowed the use of the Psychosocial Impact of Assistive Device Scale- PIADS to describe patient interaction with the Lokomat. The primary aim of this pilot study was to evaluate the psychosocial impact of the Lokomat in an in-patient rehabilitation setting using the PIADS; secondary aims were to assess whether the psychosocial impact of RAGT is different between pathological sub-groups and if the Lokomat influenced functional variables (Functional Independence Measure scale–FIM and parameters provided by the Lokomat itself). Thirty-nine consecutive patients (69% males, 54.0±18.0 years) eligible for Lokomat training, with etiologically heterogeneous walking disabilities (Parkinson’s Disease, n = 10; Spinal Cord Injury, n = 21; Ictus Event, n = 8) were enrolled. Patients were assessed with the FIM before and after rehabilitation with Lokomat, and the PIADS was administered after the rehabilitative period with Lokomat. Overall the PIADS score was positive (35.8±21.6), as well as the three sub-scales, pertaining to “ability”, “adaptability” and “self-esteem” (17.2±10.4, 8.9±5.5 and 10.1±6.6 respectively) with no between-group differences. All patients significantly improved in gait measure and motor FIM scale (difference after—before treatment values: 11.7±9.8 and 11.2±10.3 respectively), increased treadmill speed (0.4 ± 0.2m/s), reduced body weight support (-14.0±9.5%) and guidance force (-13.1 ± 10.7%). This pilot study indicates that Lokomat, in a real-world in-patient setting, may have a generalised approval, independent of disease, underlining the importance of the psycho-social framework for patients training with assistive robotic-devices.

## Introduction

In the last decade robotic devices for rehabilitation have been developed and applied to overcome disability related to walking with promising results [1–2]. Robot assisted gait training (RAGT) has been widely used in the rehabilitation of several neurologic, traumatic, vascular and neurodegenerative disorders such as Parkinson’s Disease (PD) [3], multiple sclerosis (MS) [4], Spinal Cord Injuries (SCI) [5] and Stroke [6–7], where an improvement of gait parameters and quality of life has been demonstrated.

In addition, RAGT seems not only to improve objective measurements of performance but also the perception of well-being in stroke patients [8], in SCI patients [9] and in children with cerebral palsy [10]. Although the importance of this aspect has been stated by World Health Organization (WHO) in the International Classification of Functioning, Disability and Health (ICF) as one of the three major components of disability and health [11], data on the patient-robot relationship through a psychosocial perspective are scant and, to the best of our knowledge, limited to the above mentioned reports. In particular, data are lacking in the rehabilitation setting, where RAGT is used not only to treat patients affected by cerebral lesions (stroke, cerebral palsy) and SCI, but also in PD and multiple sclerosis.

Especially, in chronic neurological diseases the psychosocial aspects are an important component of the perceived disability [12], but no data are available on the perceived well-being and psychosocial acceptance of the RAGT training.

One of the most used questionnaires for the evaluation of psychosocial impact of assistive device is the Psychosocial Impact of the Assistive Devise Scale (PIADS) [13–14]. The main purpose of this instrument is to “map” the global psychosocial impact on quality of life regarding a specific device implementation, with a selective focus on the activities of daily living (ADL). PIADS has been used to assess the psychological impact of a wide range of assistive devices [15]. Moreover, Lokomat itself can be actually considered an “assistive device for movement”, thus, as long as the patient is performing a recovery training with RAGT, the PIADS can be used to assess the psychosocial impact of this device in the rehabilitation setting.

The primary aim of the present pilot study was to evaluate the psychosocial impact of RAGT (Lokomat) in an in-patient rehabilitation setting using the PIADS. Secondary aims were to assess whether the psychosocial impact of RAGT is different across different pathological sub-groups and if RAGT impacts also on functional variables (FIM scale -Functional Independence Measure and parameters provided by the Lokomat itself).

## Materials and methods

### Procedure

The present study is an observational retrospective cross-sectional study conducted from a database of data collected prospectively during a real-world clinical practice at the Neurorehabilitation and Neurophysiopathology Unit (NFPU) of Istituti Clinici Scientifici Maugeri SpA SB—IRCCS Montescano (PV). Data were collected between 2011 and 2012. All patients entered in the study and routine clinical management data were considered. In this period a retrospective systematic analysis of all consecutive patients trained with the Lokomat was performed and their medical records reviewed retrospectively.

The study design and protocol were approved by the Institutional Review Board and by the Ethics Committee (Comitato Etico, Istituti Clinici Scientifici Maugeri, Pavia, approval # CE 2041 date 11/4/2016 chair: Carlo Pasetti) and were in accordance with the World Medical Association’s code of Ethics (Declaration of Helsinki, 1967). When admitted to our Institution, all patients signed an informed consent for the authorization to the scientific treatment of their medical records in an anonymous form.

### Data collection

**Fig 1:**
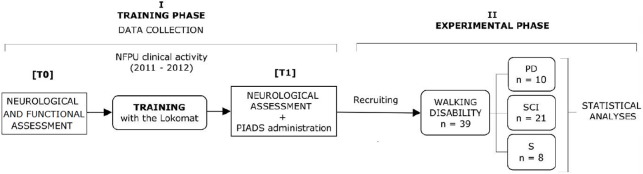
Flow diagram of the research outline.

As a routine, a neurological and functional evaluation was performed to assess patients the day before the beginning of the training with Lokomat (T0). Elegibility for treatment with Lokomat was according to Hocoma Lokomat User’s Guide, Zurich, Switzerland. The Psychosocial Impact of Assistive Device (PIADS) questionnaire was administered to 40 consecutive eligible patients who had successfully completed the training plan with the Lokomat, the day after concluding treatment (T1), to complement the whole battery of rehabilitation assessments. One patient did not agree to compile the PIADS and was therefore excluded from data analysis. All patients underwent the Functional Independence Measure (FIM scale) [16–17] assessment at the beginning and at the end of the training and comorbidity was measured with The Cumulative Illness Rating Scale (CIRS scale) [18–19]. A diagram of the research outline is shown in Fig 1

### Participants

Data from thirty-nine consecutive in-patients with PIADS (27 men, 12 females, age ranging from 33 to 79 years) with etiologically-heterogeneous walking were collected. The sample of subjects was divided a posteriori into sub-groups, depending on the aetiology underlying the motor disability. Accordingly, 3 clinical subgroups were obtained: a) first group, “Parkinson Disease” (PD, N = 10); b) second group “Spinal Cord Injuries” (SCI, N = 21, paraplegia, n = 4; paraparesis, n = 11; quadriplegia, n = 1; quadriparesis, n = 5); c) third group “Stroke Event”- dependent clinical presentations (hemiplegia, n = 5; hemiparesis, n = 3). Overall, 16 patients (41%) were not able to walk (Non-Walking) and 23 could walk (Walking).

### Instruments

#### Robot assisted gait training (RAGT)

All patients in the study underwent robot gait training with Lokomat. Lokomat is a commercially available robotic device composed of an exoskeleton, a body weight support and a treadmill. It allows facilitation of symmetric gait patterns by hip and knee components using an exoskeleton driven by programmable actuators.

At the beginning of the trial, the Lokomat parameters body weigh support and guidance force were set at the same level for all patients (50% and 100% respectively). Differently, the treadmill speed was set individually for each patient and was chosen according to the patient’s preference.

This interactive process was based on the sound patient-health care professional relationship, taking into account patient’s judgement and the need to avoid adverse events.

During the rehabilitation period, these parameters were gradually changed according to the patients’ abilities. For each patient, the training period lasted for four weeks, with 30 minutes session carried out three times a week.

#### Psychosocial Impact of Assistive Device (PIADS)

The Psychosocial Impact of Assistive Device (PIADS) questionnaire consists in a brief checklist of 26 self-report items, mostly presented as a list of familiar words or short sentences [13–14]. The different degrees of acceptance related to the assistive device are evaluated by three sub-scales. The subscale “ability” (12 items) is meant to assess the individual capability to face daily challenges and activities. The subscale “adaptability” (6 items), evaluates the willingness of taking risks and the capability to cope with different environments and new experiences. The third sub-scale, “self-esteem” (8 items), explores the psychological aspects related to mood, emotions and self-confidence, influenced by the implementation of the device in the ADLs.

The self-evaluation format is made by a 7 points Likert scale, by which the patient has to rate the degree of perceived satisfaction or improvement related to each item. The outcome score is meant to be either positive (+1, +2, +1 scores) or negative (-1, -2, -3 scores). The central tendency is marked with the zero score, defining an absence of perceived change following the device use. At the end of the measuring process, the score can be expressed through single items means, sub-scales mean or by a comprehensive scale mean. PIADS has been translated in Italian [20].

PIADS proved to have good psychometric properties [21], which were confirmed in its Italian version, being able to detect the psychosocial impact of different assistive devices [22–23].

#### Functional Independence Measure (FIM)

The Functional Independence Measure—FIM scale is a functional assessment tool widely adopted in rehabilitation as a functional outcome indicator; it measures the patient's disability level and indicates the degree of assistance required for the individual to carry out activities of daily life [16–17]. It consists of 18 items, 13 considering motor (13 items) and 5 cognitive (5 items) domains. Each FIM item is scored on a 7 point Likert scale, where one indicates complete dependence and 7 represents complete independence. The total score ranges from 18 (complete dependence on all items) to 126 (complete independence assessed in all domains). Two sub-scores can be assessed: FIM motor and FIM cognitive. All FIM evaluations were performed by certified health care professionals.

#### Cumulative Illness Rating Scale (CIRS)

The Cumulative Illness Rating Scale–CIRS [18–19] provides a comorbidity index, where clinicians rate the pathology and impairment of major organ systems of the patient. The CIRS consists of 14 health domains (hearth, respiratory system, hypertension, circulatory system, gastrointestinal apparatus, eyes-nose-throat, liver, kidney, genital-urinary tract, psychological, metabolic, neurological and musculoskeletal systems), rated from 1 (no disease, or previous problems with no sequelae) to 5 (an extremely severe problem; life threatening impairment). Scores range from 0 to 56, where zero indicates no health problems and 56 severe failure in 14 different systems (to be considered and hypothetical highest score, usually not found in clinical practice).

### Statistical analysis

Descriptive statistics for continuous variables are reported as mean ± SD. Given the relatively small number of patients for each type of disease, non-parametric statistics were used. Accordingly, between- and within-group comparisons were carried out by the Kruskal Wallis test and Wilcoxon signed-rank test, respectively. Descriptive statistics for discrete variables are reported as number (percentage frequency) and comparisons were carried out by the Chi-square test or Fisher exact test when appropriate.

A significant result for Kruskal Wallis test when comparing the patients in the three groups of disease was followed by post-hoc analysis (Dunn-Sidak), to compare pairs of groups.

To assess the effectiveness of Lokomat training, the value of each outcome variable at discharge was compared with its value at admission in the three groups.

To test whether rehabilitation effectiveness was dependent on disease type, we considered the difference between values at discharge and values at admission in the outcome variables and run a one-factor non-parametric analysis of variance (Kruskal Wallis) on the type of disease (three subgroups).

To assess the global degree of acceptance, perceived efficacy, satisfaction and psychological impact related to the use of Lokomat, PIADS scores were analysed on the whole population and across the three subgroups. The association between PIADS scores and age and type of disease was analysed by multivariable regression methods.

All statistical tests were two-tailed and statistical significance was set at p < 0.05. All analyses were carried out using the SAS/STAT statistical package, release 9.2 (SAS Institute Inc., Cary, NC, U.S.A.).

## Results

Table 1 reports demographic and baseline clinical data for the three groups of patients. Patients had similar gender distribution, education, disease duration and comorbidity (CIRS), while age was different (p<0.0001), patients with spinal cord injury being younger than the others. As far as values of FIM scale are concerned, at baseline the cognitive subscale only was different across pathologies (p = 0.019), with Stroke patients being the most compromised. Baseline parameters for Lokomat (i.e. body weight support and guidance force) were set at the same level for all patients (i.e. 50% and 100%, respectively) while treadmill speed, chosen as the best tolerated, ranged from 1.2 m/s to 1.9 m/s.

**Table 1:** Demographic and baseline clinical data for all patients and for the three different groups of patients.

Variable	ALL	PD	SCI	Stroke	p-value
Age (years)	54.0 ± 18.0	66.2 ± 6.7	43.0 ± 17.4	67.5 ± 7.6	<0.0001
Education (years)	9.3 ± 3.7	8.8 ± 3.8	9.8 ± 3.7	8.4 ± 4.0	0.60
Months of disease	43.6 ± 31.9	56.0 ± 34.2	41.0 ± 33.0	35.0 ± 24.0	0.34
Male gender (%)	27 (69)	5 (50.0)	16 (76.2)	6 (75.0)	0.31
FIM Total Score	91.0 ± 16.4	94.3 ± 20.1	92.6 ± 15.7	82.8 ± 11.7	0.28
FIM Motor	57.9 ± 15.2	62.0 ± 17.8	57.6 ± 15.6	53.8 ± 10.7	0.53
FIM Cognitive	33.1 ± 3.8	32.3 ± 3.1	35.0 ± 0.2	29.0 ± 6.0	0.0001
CIRS	12.2 ± 3.6	12.2 ± 3.9	11.2 ± 3.2	14.8 ± 3.7	0.06

ALL: overall population; PD: Parkinson’s Disease patients; SCI: Spinal Cord Injuries; FIM: Functional Independence Measure; CIRS: Cumulative Illness Rating Scale

Reported p-values are pertaining to between-group comparisons

Table 2 reports the changes in all considered variables for all patients and after stratification by type of disease. All changes in all groups were significantly different from 0 (all p values <0.01), indicating improvement, with the exception of FIM Cognitive. The last column in Table 2 reports the p-value for the between-group comparison of the changes. The only significant difference in improvement between-group was observed in the body weight support. Post-hoc analysis revealed that the only significant difference between pairs of groups was between PD and SCI (p<0.05).

**Table 2:** Changes (values after RAGT-values before RAGT) in variables for all patients and for the three different groups of patients.

	ALL	PD	SCI	Stroke	p-value
FIM Total Score	11.7 ± 9.8	13.8 ± 8.4	10.9 ± 11.2	11.3 ± 7.8	0.74
FIM Motor	11.2 ± 10.3	13.2 ± 8.3	10.2 ± 12.1	11.3 ± 7.8	0.76
FIM Cognitive	0.2 ± 0.6	0.6 ± 1.0	0.1 ± 0.5	0.0 ± 0.0	0.08
Lokomat Body Weight Support (%)	-14.0 ± 9.5	-20.0 ± 8.4	-10.7 ± 9.6	-15.1 ± 7.2	0.031
Lokomat Guidance Force (%)	-13.1 ± 10.7	-17.7 ± 7.1	-11.5 ± 12.4	-11.6 ± 8.9	0.30
Lokomat Treadmill Speed (m/s)	0.4 ± 0.2	0.3 ± 0.1	0.4 ± 0.3	0.4 ± 0.2	0.33

ALL: overall population; PD: Parkinson’s Disease patients; SCI: Spinal Cord Injuries; FIM: Functional Independence Measure.

Reported p-values are pertaining to the between-group comparisons.

All changes within all groups were significantly different from 0 (all p values <0.01), with the exception of FIM Cognitive

As far as PIADS scores are concerned, only a small minority of negative scores were reported, ranging from 0.8% for Adaptability sub-scores, to 4% for Competence sub-scores and to 7% for Self-esteem sub-scores. Table 3 reports global and adaptability, ability and self-esteem sub-scales and the p-values for between group comparisons. It can be noted that overall PIADS score and all sub-scores are around the middle of the positive range (+1,+2). No between-group differences were observed.

**Table 3:** PIADS total and subscale scores for all patients and for the three different groups of patients.

	ALL	PD	SCI	Stroke	p-value
PIADS Total score	35.8 ± 21.6	40.3 ± 24.6	34.8 ± 22.8	32.8 ± 15.1	0.74
Subscale 1: Competence	17.2 ± 10.4	19.9 ± 11.9	16.6 ± 10.8	15.5 ± 7.8	0.63
Subscale 2: Adaptability	8.9 ± 5.5	9.2 ± 6.1	8.9 ± 5.8	8.5 ± 4.8	0.97
Subscale 3: Self-esteem	10.1 ± 6.8	11.2 ± 7.5	10.1 ± 7.5	8.8 ± 4.1	0.76

ALL: overall population; PD: Parkinson’s Disease patients, SCI: Spinal Cord Injuries.

Reported p-values are pertaining to the between-group comparisons.

Moreover, no between group differences were observed between the 23 patients who could walk and the remaining 16 who could not (Table 4).

**Table 4:** PIADS total and subscale scores for walking/not walking patients.

	N	Walking	N	Not walking	p-value
PIADS Total score	23	37.7 ± 20.5	16	33.1 ± 23.5	0.52
Subscale 1: Competence	23	18.1 ± 10.3	16	15.9 ± 10.8	0.53
Subscale 2: Adaptability	23	9.2 ± 5.2	16	8.4 ± 6.1	0.65
Subscale 3: Self-esteem	23	10.3 ± 6.0	16	9.8 ± 8.0	0.79

Finally, multivariable regression analysis did not reveal any association between PIADS (global and sub-scores) and age or disease type.

## Discussion

In literature there are several important reports on the efficacy of robot assisted gait training (RAGT) on gait and other motor parameters and now it is accepted and widely applied in the rehabilitation setting of various neurological diseases (PD, MS, SCI, stroke) to improve gait [3,4,5,6,7]. However, although in rehabilitation gait and motor parameters improvement are as important as well-being and improvement in quality of life, data on well-being changes induced by RAGT are very limited and not standardized. Consequently, this lack of standard evaluation tools makes it difficult to compare the results from different researchers, stressing the need for an easy, reliable and accepted instrument for the end users [24]. This could be ascribed to the lack of specific instruments able to describe clinical aspects related to the various RAGT devices actually available in rehabilitation. Only few examples without structured questionnaires exist regarding stroke [8], SCI [9] and cerebral palsy [10]. For this reason, in the present pilot study, we evaluated the psychosocial impact of RAGT (Lokomat**®**) as delivered in a real-world hospital setting by means of the PIADS questionnaire [13–14]. PIADS has been translated in several languages as well as in Italian [20] and consists in a brief checklist of self-report items, presented in familiar words and short sentences [13–14]. These two points were considered prerequisite for its utilisation. Moreover, the third PIADS sub-scale, “self-esteem”, gives a global psychological aspects related to mood, emotions and self-confidence and has been used in a broad range of clinical contexts with different and even more sophisticated devices than Lokomat as accelerometer-triggered electrical stimulation for reaching and grasping in chronic stroke patients [25] or as eye tracking communicative device in amyotrophic lateral sclerosis [26].

In this pilot study PIADS was used to evaluate the psychosocial impact of RAGT (Lokomat) in an in-patient rehabilitation setting.

RAGT global acceptance as measured by PIADS, resulted high among the study population and the average global score ranked between +1 e +2. From a psychosocial point of view, it is worth noting that the absence of differences in global acceptance between the groups underlines that the acceptability level of RAGT as performed with Lokomat is comparable for all the diseases of the study, despite qualitative differences of each disease (i.e. motor disability level). Similarly, training perception is not different between walking and not walking patients. Probably, the device improved active patient participation and challenge, despite disease and disability; in different studies on stroke, robotic gait training showed positive effects on patients regarding psychological status and expectations [8,27]; at the same time active training is essential to optimize motor learning [28].

In our sample, systematic use of the device does not seem to constitute a disorienting experience and “fear of robots” is not observed, as described by other authors [29–30]. Moreover and partially unexpected, also age is not a limiting parameter for device acceptance [31]. These results could be due by the preliminary careful screening adopted for eligibility for treatment with Lokomat according to Hocoma User’s Guide, which excludes low level of comprehension, established as the ability of the patient to understand the Lokomat indications and functioning.

However it is notable that although the percentage of positive response on PIADS -overall PIADS score and all sub-scores are around the middle of the positive range (+1,+2)- still there is a small minority of negative scores, ranging from 0.8% for adaptability sub-scores, to 4% for competence sub-scores and to 7% for self-esteem sub-scores, underlying the possibility that not all the patients are keen to undergo RAGT. Therefore, the need to assess the psychosocial acceptance of RAGT in all patients regardless for the aetiology of the motor impairment is mandatory.

As far as the second aims of this report are concern, after RAGT training, all motor device parameters (guidance force, body weight support and treadmill speed) improved in each group, so that Lokomat training, accordingly with present literature, shows efficiency for device parameters [32,33,34,35].

Notably, the reduction in body weight support was significantly different at the end of Lokomat training between the groups; but, as expected, post-hoc analysis revealed that the only significant difference between pairs of groups was between PD (best improvement) and SCI (worst improvement, p<0.05). As reported in other studies, body weight-supported high-intensity motor training, even with no robotic device, improved gait ability in Parkinson’s disease [36]. Since gait disability in SCI patients is usually more severe than in Parkinson’s disease, robot assistance is consequently higher [3,37].

Furthermore, regarding the FIM scale, i.e. independence measure applied in our study, each population significantly improved global and motor scores in accordance with other studies in SCI, stroke and multiple sclerosis [7, 38–39]. Concerning FIM to describe disability degree, the FIM cognitive subscale is the only statistically different in T0; this result is probably due to stroke patients [40]. In the same way cognitive score of Parkinson’s disease patients is suspected, since cognitive deficit in Parkinson’s disease are not less frequent [41] even if executive functioning deficits appear not to impact on rehabilitation outcome [42].

This preliminary study has some limitations. PIADS has been applied in RAGT in a relatively small group of patients, limiting the strength of our conclusions. We also are aware that PIADS has not been originally designed for RAGT, however since no other instruments are actually available and Lokomat itself is an “assistive device for movement”, PIADS has been used and it proved to be an easy and reliable tool to assess the psychosocial impact of this device adoption in a rehabilitation setting.

So then, the lack of standard scales makes difficult to compare the results from different researchers for the subjective assessment of robot devices use, pinpointing the need for a valid and reliable instrument intended for end-users. With these recognised limitations these are, to the best of our knowledge, the first data demonstrating the positive psychosocial impact of RAGT in a real-world rehabilitation setting.

## Conclusions

In rehabilitation there is an increased need to assess if a given high technology rehabilitation procedure/intervention is useful as delivered in a real-world rehabilitation setting [24–43]. Unfortunately, standard scales to assess the psychosocial impact of RAGT are lacking, making it difficult to compare the results from different researchers on the subjective assessment of robotic devices use. In the present pilot study, we tried to overcome this limitation using the Psychosocial Impact of the Assistive Device Scale, an instrument not originally designed for RAGT. The PIADS questionnaire proved to be an easy and reliable tool to assess the psychosocial impact of RAGT in a population of patients with different walking disabilities. We found that RAGT, as delivered in an in-patient setting, may have a positive psychosocial impact to be added at the more studied and accepted gait and motor improvement. This datum confirms the importance of the bio-psycho-social framework when dealing with patients under intensive neuro-rehabilitation for motor impairments with assistive devices.

Considering the rapidly changing world, both in everyday life expectation and more specifically in high technology rehabilitation, further studies are deserved to focus on bio-psycho-social impact of robot-assisted rehabilitation. Even though the findings from this relatively small study need confirmation from larger studies, our results indicate that the PIADS seems to be an appropriate tool for subjective assessment of the psychosocial impact of RAGT, even if some specific information on RAGT could be lacking. In the light of the increasingly widespread use of high technology rehabilitation, in order to overcome the existing gap in subjective assessment of rehabilitation on assistive robot devices [24], the availability of easy and reliable instruments for the evaluation of the psychosocial impact of RAGT is mandatory. The PIADS, if associated with specifically designed instruments on RAGT impact, could provide significant information enabling clinicians to move towards a tailored and a more effective real-world rehabilitation intervention.

## Supporting information

S1 File Patients data for statistical analysis. (XLS)
